# Exploring network relations between healthcare access and utilisation in individuals with rare diseases

**DOI:** 10.1016/j.puhip.2025.100593

**Published:** 2025-02-13

**Authors:** Wehrli Susanne, Dwyer Andrew A, Matthias Baumgartner R, Landolt Markus A

**Affiliations:** a Department of Psychosomatics and Psychiatry, University Children's Hospital, University of Zurich, Zurich, Switzerland; b Division of Child and Adolescent Health Psychology, Department of Psychology, University of Zurich, Zurich, Switzerland; c Children's Research Centre, University Children's Hospital Zurich, University of Zurich, Zurich, Switzerland; d University Research Priority Program “ITINERARE –Innovative Therapies in Rare Diseases”, University of Zurich, Zurich, Switzerland; e Division of Metabolism, University Children's Hospital Zurich, University of Zurich, Zurich, Switzerland; f Boston College, William F. Connell School of Nursing, Chestnut Hill, MA, USA; g P50 Massachusetts General Hospital – Harvard Center for Reproductive Medicine Boston, MA, USA

**Keywords:** Rare disease, Healthcare utilisation, Healthcare access, Chronic disease, Network analysis

## Abstract

**Background:**

Rare diseases affect fewer than one in 2000 people and impact approximately 400 million individuals globally. High costs, uncoordinated care, and inadequate provider knowledge pose challenges to rare disease care. We aimed to examine the relationship between healthcare access and utilisation among rare disease patients in Switzerland.

**Study design:**

A cross-sectional survey was conducted with 314 individuals with a rare disease.

**Methods:**

Participants completed the Perception of Access to Healthcare Questionnaire (PAHQ) and provided data on healthcare utilisation (institutional and provider levels). Network analysis assessed nodes were based on expected influence (EI), predictability, and bridge centrality (BC).

**Results:**

Four PAHQ subscales (acceptability, availability, adequacy, and awareness) exhibited higher EI and predictability. Conversely, accessibility and affordability of healthcare services had lower EI and predictability scores. In terms of healthcare utilisation, hospitals, private practices, general practictioners (GPs), mental health professionals, and emergency services demonstrated elevated EI and predictability. Specialists and holistic healthcare providers exhibited lower EI and predictability. Affordability, disease course, as well as hospital, and GP utilisation had elevated BC values and emerged as key connectors between access and utilisation.

**Conclusion:**

This study illuminates the intricate dynamics of healthcare experiences for patients with rare diseases. This work validates network analysis as a valuable tool for examining healthcare systems. Findings can inform policies that address challenges faced by this vulnerable population, namely care integration for individuals with an unstable disease course.

## 1 What this study adds

• Demonstrates the utility of network analysis to explore the relationship between healthcare access and utilisation in rare disease populations.
• Identifies the centrality of disease progression, affordability, hospital utilisation, and GP utilisation as bridges, highlighting their role in linking access and utilisation within the healthcare network for rare disease patients and informing potential avenues for future policy and practice interventions.
• Shows that specialist and holistic care providers are poorly integrated into mainstream healthcare services for rare disease patients.

## 2 Implications for policy and practice

• The study highlights the need to develop policies to improve multidisciplinary care integration and to address challenges related to GP gatekeeping practices, which are primarily focused on cost containment.
• Improving care coordination between Swiss reference centers and European reference networks could reduce current gaps in specialised care for rare disease patients.

## 3 Introduction

Rare diseases typically result in reduced quality of life, disability, and shortened life expectancy [1,2]. There is no universal definition of what makes a disease 'rare' with global variations across regions [3]. In the European Union, a rare disease is defined as affecting fewer than 5 cases per 10,000 people [4].

Patients with rare diseases face reduced access to healthcare, longer waiting times, inadequate insurance, high out-of-pocket costs, uncoordinated care, and limited provider knowledge [5,6]. Recent studies show that they have higher healthcare utilisation and rely on multiple specialists due to the complex nature of rare diseases, increasing costs for individuals and healthcare systems [7,8]. Given their severity, patients often require lifelong healthcare for ongoing management and treatment.

Access to healthcare is a complex, multidimensional concept influenced by providers, patients, and the overall system (e.g. insurers) [9]. Historically, utilisation has been used as a proxy for access, assuming if services are used, they are accessible [10]. However, utilisation does not equate to access, as it merely indicates service use [9]. Hence, being able to use healthcare institutions and providers does not necessarily equate to comprehensive access to healthcare [11]. An exclusive focus on utilisation neglects the broader context of enabling factors and barriers thus providing an incomplete understanding of access. Conversely, solely measuring healthcare access may provide insight into potential barriers yet does not necessarily indicate whether barriers affect actual utilisation. While utilisation and access are not the same, they are interconnected with different covariates. Utilisation is affected by age, gender, education, and health insurance coverage [12]. In rare diseases, disease course and misdiagnosis impact both access and utilisation [6,13].

We aim to examine the relationship between healthcare access and utilisation of institutions and providers in a diverse Swiss rare disease sample using network analysis. Previous studies rarely examine both access and utilisation together. Network analysis visually maps complex relationships within the healthcare system, revealing dynamics of utilisation and strategies for improvement [14]. Given the unique challenges faced by rare disease populations, analysing both access and utilisation with their covariates is crucial for targeted interventions and health policy. This study has two aims. First, to determine if and how utilisation is associated with specific dimensions of access to care. Second, to identify nodes bridging access and utilisation. No specific hypotheses are formulated due to the study's exploratory nature.

## 4 Methods

### 4.1 Study design and data collection

An anonymised cross-sectional online survey was conducted among rare disease patients in Switzerland. The study followed the ethical guidelines of the Institutional and National Research Commissions and the Declaration of Helsinki of 1964 and its revisions. Informed consent was obtained from all participants. Participants were recruited through patient organisations and medical professionals at university children's hospitals in Zurich, Bern, and Lausanne, via newsletters, social media, emails, and patient advocacy groups. Eligible participants were 18 years of age or older, living in Switzerland, fluent in German, French, Italian, or English, and had a confirmed diagnosis of a rare disease [15].

Of the 606 participants, several were excluded: 4 did not give consent, 4 were not Swiss residents, 5 could not specify their disease, and 15 did not meet the rare disease definition. In addition, 229 were excluded because they did not complete the Perception of Access to Healthcare Questionnaire (PAHQ). To maintain data diversity, we did not remove outliers identified by boxplot analysis (see Additional file 2), resulting in a final dataset of 341 participants.

### 4.2 Measures

#### 4.2.1 Sociodemographic information & covariates

Respondents provided socio-demographic data including gender, age, health insurance type (public vs. private), and education level. Education was categorised as 'low' (special education or incomplete compulsory education) or 'high' (upper secondary school, high school, technical schools/seminars, or university degree) [16]. Self-reported medical diagnoses were classified using International Classification of Diseases 10th (ICD-10) and 11th (ICD-11) revision systems, chosen for their accuracy in rare disease research [17]. 22 cases were classified using the ICD-10 system instead of ICD-11 because the conditions did not exist in ICD-11 (see Additional file 4). Participants also reported their disease course (stable vs. unstable) and the number of misdiagnoses, if any. If unsure, they could select 'I don't know' or choose from ranges (1–2, 3–5, 5–10, >10), with the median of the selected range used for analysis.

#### 4.2.2 Healthcare access

We used the PAHQ to measure healthcare access. Recently adapted and validated for rare disease patients, the PAHQ did not include all 30 items of the original version [18]. Removing item 19 from the affordability subscale improved internal consistency, indicating it might represent a distinct aspect of financial barriers, while other items capture general cost issues. Given the importance of financial barriers in rare diseases, we retained this item. The adapted 27-item instrument uses a five-point Likert scale ranging from 0 ('never') to 5 ('almost always') [19]. The PAHQ assesses access to healthcare across six dimensions: acceptability ('Healthcare staff listens carefully to what I have to say. '; α = 0.92), accessibility ('Distance between healthcare facilities and my home is appropriate.'; α = 0.92), adequacy ('Expected time to receive the medical care I need is appropriate.'; α = 0.80), affordability ('To solve a health problem, I first go to a family doctor/general practitioner'; α = 0. 48), availability ('Healthcare staff is adjusted to the number of patients and their needs. '; α = 0.68), and awareness ('Information I receive is prepared in such a way that I understand it. '; α = 0.87).

#### 4.2.3 Healthcare utilisation

Assessment of utilisation was based on a previous study of a rare disease population and focused on utilisation, related to the participant's rare disease, over the past 12 months [20]. Healthcare utilisation was assessed from participants at both the institutional and the provider level. At the institutional level, we assessed the frequency of hospital visits and private practice consultations. At the provider level, we measured the use of services from specialists, general practitioners (GP), mental health professionals, emergency medicine personnel, and holistic health practitioners (including complementary medicine).

#### 4.2.4 Statistical analysis

The analysis was performed using R statistical software [21]. The present analysis is distinct from Social Network Analysis (SNA), which focuses on social entities and their interactions [22], instead our work leverages a multivariate framework to explore correlations between healthcare access and utilisation variables [23]. Covariates included age, gender, education, insurance coverage, and disease course. The number of misdiagnoses could not be included as a node due to numerous missing values diminishing the sample size to under 300 (i.e. powerly package minimum sample size required for the current study) [24]. We included six PAHQ subscales and utilisation (at both institutional and provider levels). Nodes represented variables, and edges represented partial correlations [25]. Mixed graphical models (MGMs) from the mgm package were used [26], applying LASSO to promote a sparse network and minimise false positives [27]. The qgraph package visualised the network, with green edges for positive and red for negative relationships [28]. Centrality indices were not calculated due to unreliability [29]; instead expected influence (EI) (the sum of all node edges without considering their absolute values) [30], predictability (akin to R^2^, the extent to which the variance of a node can be explained by its direct connections within the network) [31], and bridge centrality (BC) (to identify bridging nodes, connecting distinct groups within the network) using the networktools package [32], were computed. The bootnet package assessed the analysis's stability [25]. A non-parametric bootstrap generated 1000 samples to assess edge weight stability, and a case-dropping subset bootstrap (1000 samples) assessed centrality indices' stability, with a correlation stability coefficient threshold set at *r* = 0.7 or higher [23].

## 5 Results

### 5.1 Participant characteristics

Participant characteristics are shown in Table 1. The mean age of the sample was approximately 47.58 years, and around 40 % of participants were male. Around 57 % had a high level of education, 21 % were privately insured and 40 % had a stable disease course. The average number of misdiagnoses per participant was 1.62. The most common conditions identified were developmental anomalies, followed by diseases of the nervous system, and finally endocrine, nutritional or metabolic disorders.

**Table 1:** – Descriptive statistics of full sample (N = 341).

Sociodemographics	
Age, *M* (*SD*)	47.58 (15.36)
Male gender, *n* (%)	135 (40 %)
Higher education, *n* (%)	194 (57 %)
Private insurance, *n* (%)	71 (21 %)
Stable disease course, *n* (%)	135 (40 %)
Number of misdiagnoses, *M* (*SD*)	1.62 (2.61)
Unknown	76
Disease type according to ICD-11, *n* (%)	
Developmental anomalies	64 (18.8 %)
Diseases of the blood or blood-forming organs	29 (8.5 %)
Diseases of the circulatory system	8 (2.3 %)
Diseases of the digestive system	33 (9.7 %)
Diseases of the genitourinary system	1 (0.3 %)
Diseases of the immune system	13 (3.8 %)
Diseases of the musculoskeletal system or connective tissue	1 (0.3 %)
Diseases of the nervous system	64 (18.8 %)
Diseases of the respiratory system	8 (2.3 %)
Diseases of the skin	2 (0.6 %)
Diseases of the visual system	28 (8.2 %)
Endocrine, nutritional, or metabolic diseases	63 (18.5 %)
Neoplasms	5 (1.5 %)
Not categorised in ICD-11	22 (6.5 %)

PAHQ, *M* (*SD*)	

Acceptability	3.76 (0.82)
Accessibility	3.72 (1.04)
Adequacy	3.51 (0.75)
Affordability	3.50 (0.82)
Availability	3.66 (0.83)
Awareness	3.68 (0.82)

Utilisation during the last 12 months, *M* (*SD*)	

Institutional level	
Hospital	4.47 (7.54)
Private Practice	8.54 (10.74)
Provider level	
GP	6.69 (13.63)
Specialist	5.19 (8.09)
Psychologist/Psychiatrist	13.13 (11.95)
Holistic health practitioner	11.6 (11.58)
Emergency medicine personnel	1.85 (1.46)

Note: GP = General practitioner. PAHQ = Perceived access to healthcare questionnaire*. M* = mean. *N* = sample size. *SD* = standard deviation.

### 5.2 Regularised full model

**Fig. 1:**
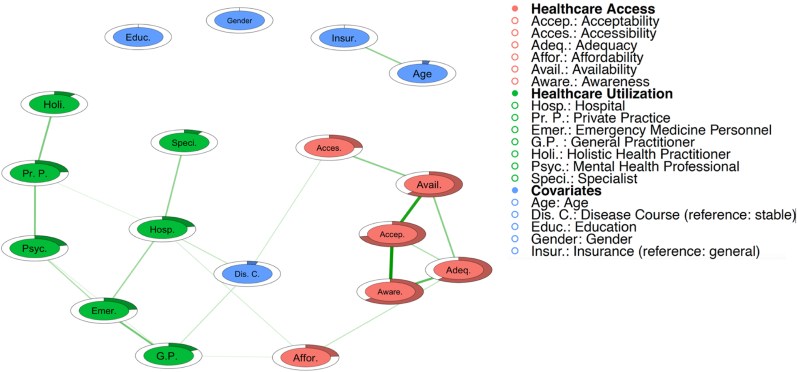
– The network illustrates the relationship between access to and utilisation of healthcare, including covariates. Green edges indicate positive associations. Rings around the nodes represent the variance within each variable, with shaded areas indicating the proportion of variance in each node that is explained by its links to other nodes.

Fig. 1 shows the positive relationships between the six PAHQ subscales and utilisation, controlling for various covariates.

A single partial correlation was found between affordability and hospital use *(r* = 0.08), representing the only direct link between access and institutional utilisation. At the provider level, the notable correlation was between affordability and GP utilisation (*r* = 0.07). Partial correlations with covariates, namely disease course, emerged with accessibility (*r* = 0.09), hospital utilisation (*r* = 0.09), and GP utilisation (*r* = 0.11). The strongest correlations were between acceptability and awareness (*r* = 0.45) and acceptability and availability (*r* = 0.40), followed by adequacy and awareness (r = 0.29). See Supplementary Table 1 for all partial correlations.

Ranking nodes from highest to lowest EI scores within the PAHQ subscales revealed acceptability, availability, adequacy, and awareness, while accessibility and affordability scored lowest. For utilisation, the highest to lowest scores at the institutional level were hospital and private practice, and on the provider level: emergency physician, GP, psychologist/psychiatrist, holistic practitioner, and specialist. Except for disease course, age, and insurance coverage, all covariates had EIs <0.100, (Table 2).

**Table 2:** – Expected influence, predictability and bridge centrality values of the network between access and utilisation of health care, including covariates.

	EI	Predictability	BC
PAHQ			

Acceptability	0.994	71.7 %	0.000
Accessibility	0.303	24.9 %	0.095
Adequacy	0.763	60.7 %	0.000
Affordability	0.267	23.3 %	0.151
Availability	0.823	61.5 %	0.000
Awareness	0.734	63.5 %	0.000

Utilisation during the last 12 months			

Institutional level			
Hospital	0.628	21.4 %	0.195
Private Practice	0.522	23.5 %	0.000
Provider level			
GP	0.489	16.9 %	0.174
Specialist	0.194	9.0 %	0.000
Psychologist/Psychiatrist	0.443	21.1 %	0.000
Holistic health practitioner	0.230	10.7 %	0.000
Emergency medicine personnel	0.567	23.3 %	0.000

Sociodemographics			

Age, *M* (*SD*)	0.195	0.000	0.000
Male gender, *n* (%)	0.000	0.000	0.000
Higher education, *n* (%)	0.000	0.000	0.000
Private insurance, *n* (%)	0.195	0.000	0.000
Stable disease course, *n* (%)	0.313	5.2 %	0.313

Note: BC = bridge centrality. EI = expected influence. GP = general practitioner.

The highest to lowest predictability values from the PAHQ subscales were acceptability, awareness, availability, and adequacy, while the lowest were accessibility and affordability. At the institutional level, private practice scored highest, followed by hospital, and at the provider level, emergency medicine personnel accounted for the highest predictability followed by psychologists/psychiatrists, GPs, holistic practitioners, and specialists. Covariates generally showed low predictability, all below 1 %, except for disease course.

Disease course had the highest bridge centrality, followed by hospital and GP utilisation, and affordability; other variables had no noteworthy values.

### 5.3 Stability analyses

The bootstrap analysis of edge weights confirmed the network model's high accuracy. Stability analysis for centrality produced a CS-coefficient of 0.751, indicating high stability [23]. Similarly, edge weight stability yielded an ES-coefficient of 0.751 and a bridge strength coefficient of 0.129, both considered stable [23,31]. See Supplementary Figs. S2 and S3 for results.

## 6 Discussion

This work aimed to enhance our understanding of the complex relationship between healthcare access and utilisation in the context of rare diseases. We sought to explore the relationship between provider and institutional utilisation with dimensions of access. Our second aim was to identify points bridging access and utilisation using network analysis. The results demonstrate a consistent positive correlation between the PAHQ subscales and healthcare utilisation, with disease course identified as a significant covariate. The node of acceptability, emerged as the most influential, followed by availability, adequacy, and awareness. This observation highlights the importance of establishing a therapeutic relationship and person-centered care for patients with rare diseases. In contrast, the variables of accessibility and affordability demonstrated less influence. In the context of healthcare providers, private practices, hospitals, emergency medicine, GPs, and psychologists/psychiatrists exhibited the highest levels of EI, whereas holistic practitioners and specialists demonstrated lower levels of predictability and EI. Additionally, disease course exhibited a notable level of EI and predictability, although this was less pronounced than the variables pertaining to access and utilisation. The bridge centrality analysis revealed that disease course, hospital and GP utilisation, and affordability play a pivotal role in connecting access to utilisation.

Our findings indicate that access dimensions including acceptability (service matches provider's preference), availability (adequate resources), adequacy (service hours, referral systems), and awareness (effective patient communication) had the highest predictability, highlighting their interdependency as emphasized in previous theoretical models of access [9,33,34]. Conversely, lower predictability for accessibility (proximity in time and distance) and affordability (costs) might be due to external factors, notably policy impacts. In Switzerland, non-progressive premiums and high out-of-pocket costs disproportionately affect lower income individuals [35]. Our study included data on insurance coverage but did not account for factors such as subsidies for low-income households, the role of cantonal (i.e. state/province) health governance, or geographic considerations affecting healthcare accessibility (i.e. remote, underserviced regions), which are all systemic aspects of access not included in the theoretical models this paper is derived from Ref. [36]. Utilisation variables had higher predictability than accessibility and affordability but were lower than other access dimensions. These observations align with literature suggesting access and utilisation are distinct constructs influenced by different factors [9,10].

Notably, acceptability, availability, adequacy, and awareness showed the highest EI, highlighting their key role in healthcare access and utilisation patterns [9]. These findings provide further evidence that patient perceptions significantly influence healthcare utilisation, especially in rare diseases where provider knowledge may be low and past negative or stigmatising experiences can reduce utilisation [6]. Observed higher EI scores for mental health professionals, GPs, and emergency physicians reflect their integral role within the care process. However, specialists and alternative care services had lower EI, indicating a need for better integration into mainstream care for the population in question, particularly for rare disease patients who frequently necessitate holistic approaches, due to the lack of available treatment options [37]. Integrating services through interdisciplinary collaboration and improved referral systems could enhance accessibility, utilisation, and patient satisfaction [38]. Moreover, addressing the gatekeeping role of GPs, who frequently serve as the primary point of contact for referrals to specialists, can provide additional support for these improvements, as discussed in greater detail below.

We also sought to identify points bridging access and utilisation using network analysis. Bridge centrality analysis revealed the crucial contributions of an unstable disease course, GP utilisation, hospital utilisation, and affordability in linking access and utilisation. The high bridge centrality of unstable disease course (characterised by unknown, progressive, or episodic patterns), aligns with the need for frequent healthcare visits [13] contributing to increased costs, thus reinforcing the linkage between affordability and utilisation [8]. GPs and hospitals likely exhibited bridge centrality due to their critical roles in healthcare delivery: GPs often serve as the first point of contact for patients, facilitating access to specialised care, while hospitals act as hubs for multidisciplinary teams and referrals. In rare diseases, centralised and multidisciplinary hospital teams improve patient satisfaction and care coordination therby promoting efficient healthcare delivery [6,13]. The observed correlations between affordability and both GP utilisation and hospital utilisation underscore how financial considerations shape care-seeking behavior. Our findings suggest that affordability connects these pivotal providers within the network, creating a reinforcing cycle where financial considerations could influence decisions to seek care. On the other hand, integrated care models offer a potential solution to mitigate the systemic barriers highlighted by affordability's role in access and utilisation. As GPs are typically more affordable than specialists [38], GPs play a central role in the Swiss healthcare system for both primary care provision and for their gatekeeping role for accessing specialised care. On the other hand, integrated care models promote proactive and structured interactions between providers, patients, and insurers, mitigating the risks of care fragmentation and improving overall patient outcomes [39]. In integrated care models, GPs acting as coordinators of medical care, a key difference from traditional gatekeeping models. In integrated care models, GPs are dedicated to coordinating different levels of care ranging from ambulatory clinical settings to inpatient hospitalisations – also known as vertical integration. A recent retrospective Swiss cohort study of chronic disease patients found that integrated care models have lower hospitalisation rates and healthcare costs compared to standard care [40]. Therefore, policy supporting integrated care models that leverage GP gatekeeping may contribute to long-term patient wellbeing and system efficiency for rare diseases.

The results of the study indicate that gaps in care coordination remains a significant barrier. The lack of integration between Swiss reference centers and European Reference Networks (ERNs) exacerbates these problems, as Swiss patients are unable to access the level of coordinated care and specialised expertise available through ERNs [41]. ERNs facilitate cross-border collaboration and connect healthcare providers and researchers to provide specialised diagnosis and treatment for rare diseases across Europe [42]. Better integration of Swiss reference centers with ERNs could address the lower EI of specialists and holistic medicine professionals by providing opportunities for collaborative care models and improved referral systems of international expertise. Additionally, ERNs could enhance the bridge centrality roles of GPs and hospitals by streamlining collaboration between primary care providers and centers of expertise. The high bridge centrality of GPs and hospitals reflects their pivotal role in linking access dimensions to utilisation. However, ERNs, through their network of specialised clinical teams and hospitals, can strengthen these links by creating direct pathways to multidisciplinary expertise for patients requiring advanced care. This integration reduces the reliance on traditional gatekeeping by ensuring that non-specialised hospitals and GPs serve as facilitators that connect patients to centers of expertise, without creating unnecessary boundaries, ultimately enhancing care coordination and patient outcomes. This direct connection to specialised care not only has the potential to improve patient outcomes but may also promote affordability by optimizing resource allocation and reducing inefficiencies by reducing the need for repeated back-and-forth interactions between GPs, hospitals, and patients. To address these gaps, it is essential that Swiss centers of reference develop stronger links with ERNs, either through formal membership or through informal collaboration and knowledge sharing. This integration would reduce the burden on patients and families and ensure that Swiss rare disease care is aligned with the latest European standards and innovations.

A limitations of this study is its cross-sectional design, which limits the identification of causality and temporal relationships. Future research employing longitudinal designs could help elucidate causality and directionalits. Our analysis relied solely on patient perspectives aligning with the focus of the theoretical models underpinning this study [9,33,34]. However, incorporating additional frameworks, such as the behavioral-ecological model [43], alongside broader data sources—such as census information or insurance records could enhance predictability and deepen our understanding of systemic factors, thereby providing valuable insights to inform policy development. Finally, the participant sample may not be representative of all rare diseases, as some diagnoses may be over or underrepresented.

In conclusion, our findings enhance the understanding of the complex relationships between access and utilisation in the context of rare diseases. By providing insight into the dynamics of access and utilisation, findings highlight the need for integrated strategies and expanded theoretical models for rare disease care. The present work supports further research and policy development to improve the healthcare experience of people with rare diseases and promote a more inclusive and efficient healthcare systems.

## Ethical approval

Ethics approval was not required for this study as data collection was conducted entirely anonymously. The Cantonal Ethics Committee of Zurich confirmed through a clarification of responsibility that the project did not fall under the scope of the Human Research Act and thus did not need formal approval. The use of patient data adhered to the ethical guidelines of the Institutional and National Research Commissions, as well as the 1964 Declaration of Helsinki and its subsequent revisions. Informed consent was obtained from all participants involved in the study.

## Authors’ contributions

SW conceived the study, was involved in the design and coordination of the study, carried out data collection, analyzed the data, and drafted the manuscript. AD conceived the study and critically reviewed the manuscript. MB conceived the study and critically reviewed the manuscript. ML conceived the study, was involved in the design of the study, supervised the data analyses, and revised the manuscript. All authors contributed to the article and approved the submitted version.

## Data availability

The data is available upon reasonable request.

## Funding

This work was supported by the University Research Priority Program of the University of Zurich (URPP) ITINERARE – Innovative Therapies in Rare Diseases, Switzerland. http://www.itinerare.uzh.ch.

## Declaration of interest statement

Wehrli, Susanne, Declarations of interest: none.

Dwyer, Andrew A., Declarations of interest: none.

Matthias Baumgartner R., Declarations of interest: none.

Landolt, Markus A., Declarations of interest: none.
