# Supplementary material for: Exploring network relations between healthcare access and utilisation in individuals with rare diseases
Source: Public Health Pract (Oxf). 2025 Feb 13;9:100593. doi: 10.1016/j.puhip.2025.100593 (PMC11929058; doi:10.1016/j.puhip.2025.100593)
Supplement: Multimedia component 1 [file mmc1.docx]

**Supplementary material**

**Table S1:** Existing partial correlation

|  |  | Partial correlations |
| --- | --- | --- |
| Acceptability | Adequacy | 0.15 |
| Adequacy | Affordability | 0.12 |
| Acceptability | Availability | 0.40 |
| Accessibility | Availability | 0.21 |
| Adequacy | Availability | 0.21 |
| Acceptability | Awareness | 0.45 |
| Adequacy | Awareness | 0.29 |
| Affordability | Hospital | 0.08 |
| Hospital | Private practice | 0.07 |
| Hospital | Emergency medicine personnel | 0.17 |
| Affordability | General practitioner | 0.07 |
| Emergency medicine personnel | General practitioner | 0.25 |
| Private practice | Holistic health practitioner | 0.23 |
| Private practice | Mental health professional | 0.23 |
| Emergency medicine personnel | Mental health professional | 0.15 |
| General practitioner | Mental health professional | 0.07 |
| Hospital | Specialist | 0.19 |
| Accessibility | Disease course (reference: stable) | 0.09 |
| Hospital | Disease course (reference: stable) | 0.11 |
| General practitioner | Disease course (reference: stable) | 0.11 |
| Age | Insurance (reference: general) | 0.19 |

**Figure S2.** Accuracy and stability of the expected influence by estimating network models based on subsets of the data.


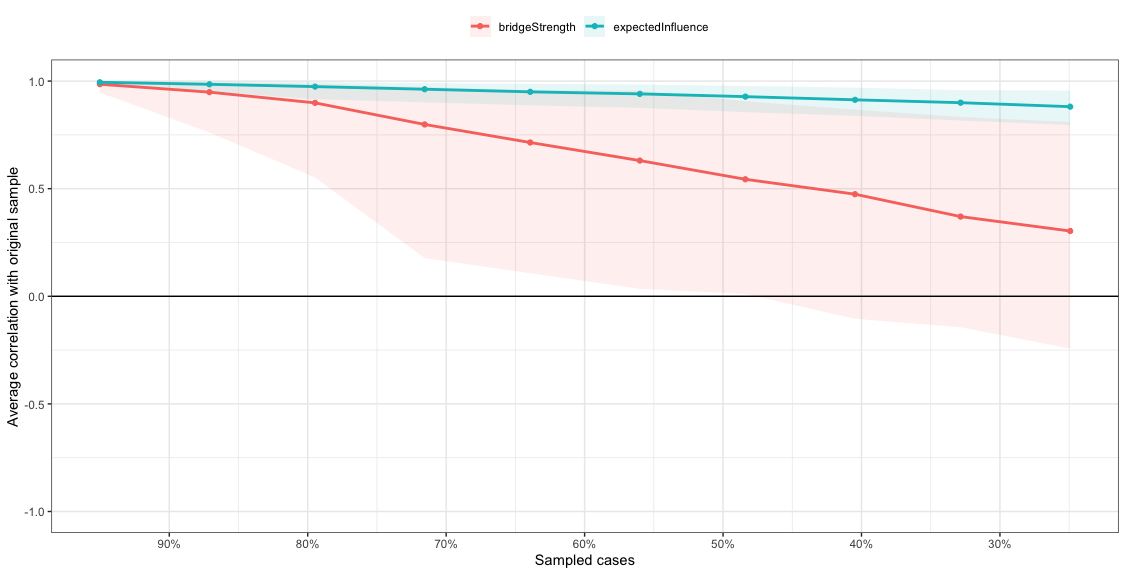


Notes: Figure visualises the accuracy and stability of EI and BC by subsetting bootstrap data showing average correlations between centrality indices between the estimated network based on the full dataset and networks estimated on subsamples.

**Figure S3.** 95% confidence intervals around the estimated edge weights


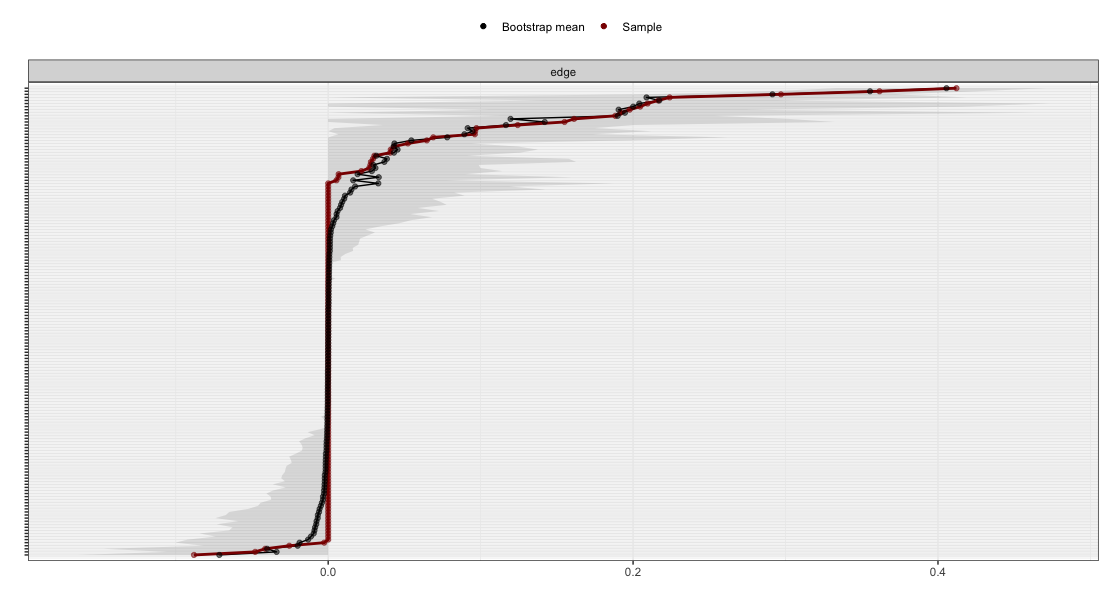


Notes: The red line indicates the edge weight values and the gray area the bootstrapped 95% CIs. Each *horizontal line* represents one edge of the network, ordered from the edge with the highest edge-weight to the edge with the lowest edge-weight.
